# Cortical Reshaping and Functional Recovery Induced by Silk Fibroin Hydrogels-Encapsulated Stem Cells Implanted in Stroke Animals

**DOI:** 10.3389/fncel.2018.00296

**Published:** 2018-09-06

**Authors:** Laura Fernández-García, José Pérez-Rigueiro, Ricardo Martinez-Murillo, Fivos Panetsos, Milagros Ramos, Gustavo V. Guinea, Daniel González-Nieto

**Affiliations:** ^1^Center for Biomedical Technology, Universidad Politécnica de Madrid, Madrid, Spain; ^2^Departamento de Ciencia de Materiales, Escuela Técnica Superior de Ingenieros de Caminos, Canales y Puertos, Universidad Politécnica de Madrid, Madrid, Spain; ^3^Biomedical Research Networking Center in Bioengineering Biomaterials and Nanomedicine, Madrid, Spain; ^4^Department of Translational Neuroscience, Instituto Cajal – Consejo Superior de Investigaciones Científicas, Madrid, Spain; ^5^Neurocomputing and Neurorobotics Research Group, Faculty of Biology and Faculty of Optics, Universidad Complutense de Madrid, Madrid, Spain; ^6^Neural Plasticity Research Group, Health Research Institute of the Hospital Clínico San Carlos, Madrid, Spain; ^7^Departamento de Tecnología Fotónica y Bioingeniería, Escuela Técnica Superior de Ingenieros de Telecomunicación, Universidad Politécnica de Madrid, Madrid, Spain

**Keywords:** brain remapping and plasticity, sensorimotor recovery, stroke, mesenchymal stem cells, silk fibroin hydrogels, somatosensory and motor cortex

## Abstract

The restitution of damaged circuitry and functional remodeling of peri-injured areas constitute two main mechanisms for sustaining recovery of the brain after stroke. In this study, a silk fibroin-based biomaterial efficiently supports the survival of intracerebrally implanted mesenchymal stem cells (mSCs) and increases functional outcomes over time in a model of cortical stroke that affects the forepaw sensory and motor representations. We show that the functional mechanisms underlying recovery are related to a substantial preservation of cortical tissue in the first days after mSCs-polymer implantation, followed by delayed cortical plasticity that involved a progressive functional disconnection between the forepaw sensory (FLs_1_) and caudal motor (cFLm_1_) representations and an emergent sensory activity in peri-lesional areas belonging to cFLm_1_. Our results provide evidence that mSCs integrated into silk fibroin hydrogels attenuate the cerebral damage after brain infarction inducing a delayed cortical plasticity in the peri-lesional tissue, this later a functional change described during spontaneous or training rehabilitation-induced recovery. This study shows that brain remapping and sustained recovery were experimentally favored using a stem cell-biomaterial-based approach.

## Introduction

Stroke represents the leading cause of disability and a main reason for premature mortality worldwide (Benjamin et al., 2017). The early recognition of symptoms and the rapidity of medical intervention influence the clinical evolution of each patient. In ischemic stroke, the most frequent form of stroke, the intravenous injection of the tissue plasminogen activator (tPA) and surgical procedures such as endovascular thrombectomy are currently the main advanced treatments for the early reestablishment of blood flow in the occluded vessel (Saver et al., 2015). However, a minority of stroke patients can really get benefits from these treatments due to the narrow time window for administration after the onset of symptoms and the risks of complications such as intracranial hemorrhage, major systemic hemorrhage, and angioedema. Thus, most of the patients who do not receive acute reperfusion therapies show long-term disabilities. Unfortunately, we do not have therapies to target the subacute and chronic phases of ischemic stroke and efficiently repair the damaged brain promoting a satisfactory degree of functional recovery in most patients (George and Steinberg, 2015).

A well-established observation in different species, including humans, is that the brain reorganizes itself under physiological and pathological conditions (Merzenich et al., 1984; Jenkins et al., 1990; Panetsos et al., 1995; Nudo et al., 1996b; Elbert et al., 1997; Traversa et al., 1997; Jaillard et al., 2005; Schaechter et al., 2006; Brown et al., 2009). During normal brain development, the emerging circuitry is organized to represent sensory, motor and variably distributed cognitive abilities. However, the initially established topographic and functional representations are not static and change dynamically in size and location throughout adult life in response to sensory input, experience and learning (Jenkins et al., 1990; Elbert et al., 1995; Nudo et al., 1996a). Brain reorganization also occurs after central or periphery injury (Merzenich et al., 1984; Calford and Tweedale, 1988; Elbert et al., 1997; Simoes et al., 2012). Reciprocal GABA-mediated inhibitory signals between distinct representations (Jacobs and Donoghue, 1991; Jones, 1993) and the regular activity of sensory/motor fields together with the maintenance of axonal pathways (Graziano and Jones, 2009) have been proposed to contribute to the mechanism defining topographic areas and preventing the functional invasion between surrounding representations (Calford and Tweedale, 1988; Jacobs and Donoghue, 1991).

The general principles that orchestrate the nascent brain circuitry during development are believed to be similar to those forming the compensatory circuits underlying functional recovery after brain damage. Based on accumulating evidence, motor and sensory representations are modified in the affected and non-affected hemispheres after unilateral brain damage (Nudo et al., 1996b; Brown et al., 2009; Harrison et al., 2013; Tennant et al., 2015). An evolutionarily conserved program exists in mammals to sustain the reorganization of non-affected areas surrounding the damaged regions that then perform the specific functions that were lost and initially depended of the injured areas. However, under spontaneous recovery, brain remapping in the undamaged surrounding tissue does not always correlate with functional outcomes (Nudo and Milliken, 1996; Xerri et al., 1998; Schaechter et al., 2006; Nishibe et al., 2015). A direct link between the post-stroke cortical changes and the temporal pattern of functional recovery has not been directly obtained in the majority of the studies, making a cause-effect relationship difficult to establish.

Stem cell therapy constitutes a promising approach to stimulate functional recovery after stroke (Chen et al., 2001a,b; Trounson and McDonald, 2015; Wang et al., 2016). Different types of stem cells have been used as a potential source of both replacement cells and neurotrophic factors although the precise mechanisms of action and the optimal administration route are unclear (Hermann et al., 2014; Gervois et al., 2016). Compared with the systemic delivery (Prasad et al., 2014; Hess et al., 2017), cerebral implantation requires fewer cells and provides a precision graft (Yang et al., 2011; Du G. et al., 2014; Du S. et al., 2014; Borlongan, 2016). However, the cerebral route has also reached relatively modest levels of post-stroke functional recovery, which has been associated with the severe loss of grafted cells that are generally not observed in the brain for more than 1–3 weeks after transplantation, as reported in several preclinical models (Kelly et al., 2004; Bliss et al., 2010; Mora-Lee et al., 2012; George and Steinberg, 2015; Wang et al., 2016). In patients, the cerebral implantation of stem cells has been reported to be safe and clinical improvements were observed, but the small sample size and heterogeneity between subjects currently preclude the use of this specific approach in clinical practice (Chen et al., 2014; Borlongan, 2016; Steinberg et al., 2016).

The use of biomaterials in tissue engineering is booming and has provided examples of how the integration of neurotrophic cells and factors in biomaterial-based polymers results in better post-stroke functional recovery compared to the implantation of therapeutic cells or factors alone (Guan et al., 2013; Jendelova et al., 2016; Nih et al., 2016). Different natural and synthetic polymers have been used to support stem cell engraftment including hyaluronic acid, collagen, hyaluronan-methylcellulose, polyethylene glycol, PLGA, alginate and matrigel among others (González-Nieto et al., 2018).

Although improved functional outcomes have been achieved in the majority of stroke models after the implantation of stem cells or stem cells plus different biomaterials, the mechanisms of recovery are in most cases unclear, but they might be associated with the reestablishment of the destroyed circuitry in damaged regions (Taguchi et al., 2004; Ramos-Cabrer et al., 2010; Wang et al., 2016). However, other tentative benefits of stem cell therapy could be related to the reorganization of the pre-existing circuitry in peri-lesional areas of the affected hemisphere, involving the reorganization of cortical maps (Dijkhuizen et al., 2001; Brown et al., 2009; Andres et al., 2011).

In the present study, we examined the ability of bone marrow mesenchymal stem cells (mSCs) to enhance functional outcomes after cortical stroke in mice. The mSCs were intracerebrally implanted together with a silk fibroin (SF)-based hydrogel (Fernandez-Garcia et al., 2016). We report evidence of cortical plasticity linked to functional recovery occurring late after treatment. To our knowledge, this study is unique because it shows that brain remapping and sustained recovery were experimentally favored using a stem cell-biomaterial-based approach.

## Materials and Methods

Other methods are found in the **Supplementary Material**.

### Animals

*In vivo* therapy experiments were conducted using adult male CD-1 mice (35–40 g body weight; 8–10 weeks old). For tracking studies EGFP-expressing mSCs were obtained from adult C57BL/6-Tg(CAG-EGFP)C14-Y01-FM131Osb mice, which were generously donated by Professor Masaru Okabe (Osaka University). All mice were bred and housed in the animal facility of the Center for Biomedical Technology. Animals were housed with free access to food and water in an animal room with a controlled temperature and a natural light cycle. Daily routines were performed between 8 a.m. and 5 p.m. by authorized personnel. All procedures were performed under the Spanish Regulations for animal experimentation and use of genetically modified organisms (Laws 53/2013, 178/2004 and ECC/566/2015) with the approval of Community of Madrid (PROEX 393/15) and according to the ARRIVE (Animal Research: Reporting *In Vivo* Experiments) guidelines.

### Silk Fibroin Extraction and Preparation of the Hydrogel

Silk fibroin was extracted from *Bombyx mori* cocoons, which were provided by J. L. Cenis (IMIDA, Murcia, Spain), and processed using a previously described method (Fernandez-Garcia et al., 2016). Briefly, cocoons were cut into small pieces and degummed to remove sericin. After degumming, fibroin fibers were repeatedly rinsed with distilled water and allowed to dry overnight. Dry fibers were dissolved in a lithium bromide solution under continuous stirring. The solution was dialyzed against water, subsequently centrifuged to remove impurities, frozen at -80°C and lyophilized to obtain a final SF powder. For delayed *in situ* gelling the SF solutions (2% fibroin in PBS) were sonicated using a Branson 450 Sonifier coupled to a 3 mm diameter Tapered Microtip (Fernandez-Garcia et al., 2016).

### Striatal Injection of mSCs and Silk Fibroin Hydrogels

Animals were anesthetized with ketamine (100 mg/kg) and xylazine (10 mg/kg) prior to the stereotaxic injection of phosphate-buffered saline (PBS at pH = 7.4), a SF solution in the pre-gel state, and mSCs resuspended in PBS (control) or in SF (pre-gel state). For analgesia, 0.1 mg/kg of buprenorphine was subcutaneously injected prior to and 8 h after surgery. The mice were secured in a stereotaxic frame (David Kopf Instruments, Los Angeles, CA, United States) and a skin incision was made, the skull was exposed, and a burr hole was drilled with a 0.8 mm drill bit. The rectal temperature was maintained at a constant value of 35–37°C using a heating pad. The different solutions were infused unilaterally into the caudate putamen (striatum) of the infarcted hemisphere using a Hamilton syringe with a needle (model 701SN, 31-G/Pst3, Teknokroma). Approximately 1 × 10^5^ mSCs in PBS solution or in SF solution for a total volume of 5 μl were injected (1 μl/min) at the following coordinates from bregma: posterior +1.0 mm, laterally +2.7 mm, and ventrally +2.3 mm. After injection, the needle was left in place for 5 min to allow the solution to diffuse into the striatum.

### Surgical Implantation of Electrodes and Recordings of Evoked Potentials

Evoked potentials (EPs) were recorded as described in our previous study (Barios et al., 2016). Briefly, ketamine/xylazine-anesthetized mice were implanted with two microelectrodes in the cortex of each hemisphere (±2.0 mm lateral and -0.5 mm rostrocaudal from bregma, at a depth of 0.5 mm) to record the activity of the forelimb somatosensory (FLs_1_) representation. Two additional electrodes were implanted in both hemispheres to examine the activity of the forelimb motor (cFLm_1_) representation (±1.0 mm lateral and +0.5 mm rostrocaudal from bregma, at a depth of 0.5 mm). These coordinates were based on anatomical and functional studies in mice (Paxinos and Franklin, 2004; Ferezou et al., 2007; Tennant et al., 2011; Hira et al., 2013a, 2015; Lim et al., 2014; Alia et al., 2016). Finally, two screws were placed over the visual cortex of both hemispheres and served as the indifferent and ground electrodes (±2.0 mm lateral and -3.0 mm rostrocaudal from bregma). Recordings were obtained from both hemispheres and cortical areas after forelimb contralateral electrical stimulation. Cortical signals were amplified (×10^3^) and filtered (bandpass, 10–2.000 Hz) using a portable electromyography (EMG)-evoked potentials (EPs) device (Micromed, Mogliano Veneto, Treviso, Italy).

### Statistics

All statistical analyses were conducted using SigmaPlot (Systat, Germany). Data are expressed as the means ± standard errors of the means (SEM). The significance of the differences between groups was determined using several statistical models. In most studies, an analysis of variance (ANOVA) was performed to test the differences between two or more means and contrast the null hypothesis. When the result was statistically significant (null hypothesis rejection), other statistical tests were used (Tukey’s test) to determine which groups showed significant differences. For example, a one-way ANOVA test was applied to examine significant differences in total cortical tissue loss and total subcortical inflammation. A two-way ANOVA was applied to determine the statistical significance of differences in forepaw footslips, evoked amplitude potentials, and latencies, as well as the number of EGFP-positive cells or amount of cortical tissue loss and subcortical inflammation along the rostrocaudal axis, considering time, treatment or brain section as independent variables. When applicable, statistical significance was assessed by performing Student’s *t*-test for independent samples (for example to examine evoked cortical responses between distinct hemispheric areas). Correlations between variables were determined using Pearson’s product-moment correlation coefficient.

## Results

### Somatosensory and Motor Responses in the Non-modified and Injured Mouse Brain

**FIGURE 1:**
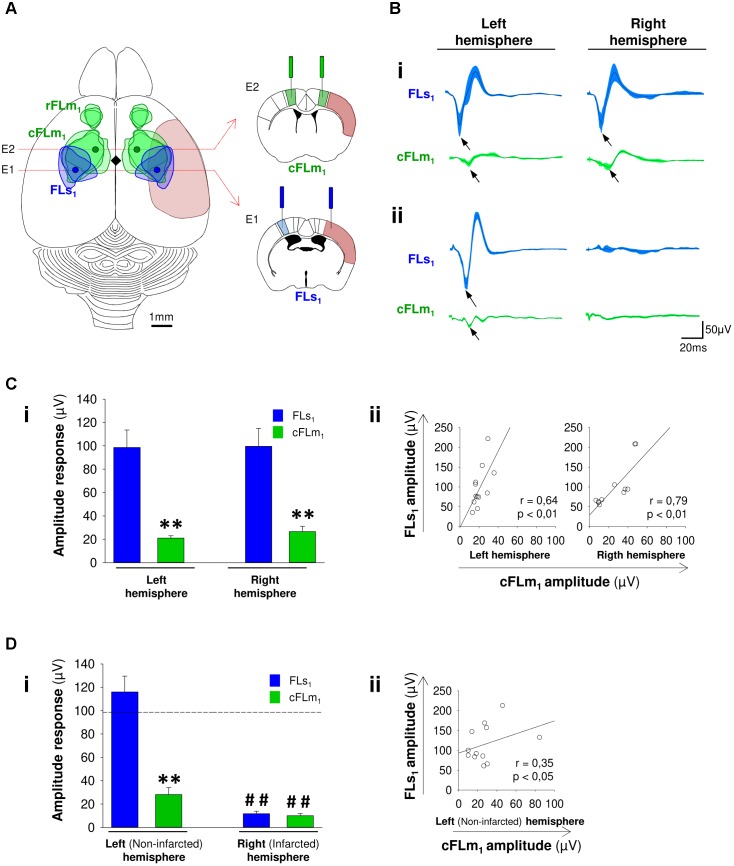
Post-stroke impairments in the functions of somatosensory and motor cortex territories. **(A)** Schematic showing the dorsal view of a mouse brain and the motor (green, FLm_1_) and the somatosensory (blue, FLs_1_) forelimb map territories based on intracortical microstimulation (ICMS) and voltage-sensitive dye imaging (VSD) studies performed in the mouse (Ferezou et al., 2007; Tennant et al., 2011; Hira et al., 2013a, 2015; Lim et al., 2014; Alia et al., 2016). In rodents, the forelimb motor cortex comprises a rostral (rFLm_1_) and a caudal (cFLm_1_) region. The cFLm_1_ region partially overlaps topographically with the somatosensory forelimb area FLs_1_. In the schematic, the soft red area represents the infarcted territory produced using the dMCAO approach that mostly affects FLs_1_ and partially affects cFLm_1_. The blue and green circles represent the site of electrodes implantation with respect to bregma (black diamond) that were used to record the evoked activity of discrete regions in FLs_1_ (infarcted site) and cFLm_1_ (tissue surrounding the lesion). Right panels depict a complementary scheme showing the position of electrodes in forelimb somatosensory and motor cortices of coronal brain sections (Paxinos and Franklin, 2004). **(B)** Evoked activity in FLs_1_ and cFLm_1_ after forelimb stimulation. Recordings are shown for both hemispheres under basal conditions (panel **i**) or after stroke (panel **ii**). In each trace the black line represents the average recordings obtained from 12 different mice. The blue or green shaded areas represent the standard errors of the means (SEM) for FLs_1_ and cFLm_1_ responses. **(C, D)** Amplitude of responses in FLs_1_ and cFLm_1_ for the left and right hemisphere under non-stroke (**C**, panel **i**) and stroke (**D**, panel **i**) conditions. The dashed black line in **(D)** represents the average amplitude of evoked potentials from FLs_1_ in both hemispheres before stroke. Pre-stroke (**C**, panel **ii**) and post-stroke (**D**, panel **ii**) Pearson’s correlation coefficients between the maximal responses in FLs_1_ and cFLm_1_. Data are presented as the means ± the SEM from 12 mice. Asterisks denote significant differences between FLs_1_ and cFLm_1_ responses. The hashed symbols indicate differences in pre- and post-stroke responses for each hemisphere (Student’s *t*-test; ^∗∗^*p* < 0.01; ^##^*p* < 0.01).

Before determining the effect of the therapy itself, we characterized the electrophysiological activity in discrete regions of the somatosensory and motor cortices in non-damaged mice in response to forelimb stimulation. We performed simultaneous recordings of evoked activity in areas of the forelimb primary-somatosensory cortex (FLs_1_, E1) and the most caudal forelimb-motor representation (cFLm_1_, E2). These specific electrode locations (**Figure 1A**), which are based on known anatomical and functional landmarks in mice and correspond to FLs_1_ (Ferezou et al., 2007; Hira et al., 2013a; Lim et al., 2014; Vanni and Murphy, 2014) and cFLm_1_ (Tennant et al., 2011; Hira et al., 2013a,b), were chosen to identify subsequent changes in infarcted (FLs_1_, E1) and surrounding peri-infarcted (cFLm_1_, E2) tissues, according to the particular stroke model used in the present study (**Supplementary Figure S1**). With this cortical stroke model, the infarcted area extended throughout the FLs_1_ representation and partially into the cFLm_1_ area (**Figure 1A** and **Supplementary Figure S1**), mostly because in mice, part of cFLm_1_ topographically overlaps with FLs_1_ (Ferezou et al., 2007; Tennant et al., 2011; Hira et al., 2013a,b).

In pre-stroke conditions, a marked response of negative polarity was detected by the electrode (E1) placed in the contralateral FLs_1_ area in response to forelimb stimulation (**Figures 1Bi,C**) with maximal response amplitude occurring at approximately 17 ms after stimulation (for the right hemisphere: 17.2 ± 0.8 ms; for the left hemisphere: 17.3 ± 0.9 ms). In the same hemisphere, a small amplitude potential was concurrently detected by the electrode placed at the cFLm_1_ representation (**Figures 1Bi,C**), with the maximal response occurring at approximately 21 ms after stimulation (for the right hemisphere: 20.7 ± 0.8 ms; for the left hemisphere: 21.2 ± 0.9 ms). A significant difference in the latency of the responses between FLs_1_ and cFLm_1_ was determined for the right (3.5 ± 0.6 ms; *p* < 0.01) and left (3.9 ± 0.8; *p* < 0.01) hemispheres. This latency value (≥3.5 ms) and the anatomical location of both electrodes were consistent with the finding that cFLm_1_ activity is dependent on the previous response in FLs_1_. Consistent with the presence of an intracortical associative pathway between FLs_1_ and cFLm_1_, the maximal amplitude of recorded responses in FLs_1_ and cFLm_1_ positively correlated in each hemisphere analyzed (**Figure 1C**). This finding is consistent with the results of previous studies showing that sensorimotor transformation is sustained by functional communication between the primary somatosensory barrel and whisker motor cortices in rats and mice (Farkas et al., 1999; Ferezou et al., 2007; Matyas et al., 2010), or between the somatosensory and motor forepaw representations after photostimulation in mice (Lim et al., 2014). In our study, the cFLm_1_/FLs_1_ ratio, which was estimated from the maximal amplitude of responses in each recorded region, was 26.2 ± 3.4 and 24.8 ± 2.8 for the right and left hemispheres, respectively (**Figure 1C**). This ratio might represent to some extent the degree of functional connectivity between the recorded areas in cFLm_1‘_ and FLs_1,_ respectively.

Cerebral infarction was induced in these mice by occluding the distal middle cerebral artery (MCA). This is a preferable stroke model that has been frequently used to mimic human strokes that do not spontaneously reperfuse or are refractory to treatment (Albers et al., 2011). One week after stroke, the animals showed a strong loss of cortical responsiveness in both the FLs_1_ and cFLm_1_ areas of the infarcted right hemisphere after forelimb stimulation (**Figures 1Bii,D**). In the non-infarcted hemisphere, a similar ratio value (26.78 ± 4.86) was obtained with respect to the pre-stroke condition, with significant correlation for the responses between cFLm_1_ and FLs_1_ (**Figure 1D**).

### Changes in Cortical Function and Connectivity Are Linked to Functional Recovery After Treatment With mSCs Delivered via Silk Fibroin Hydrogels (mSCs-SF)

**FIGURE 2:**
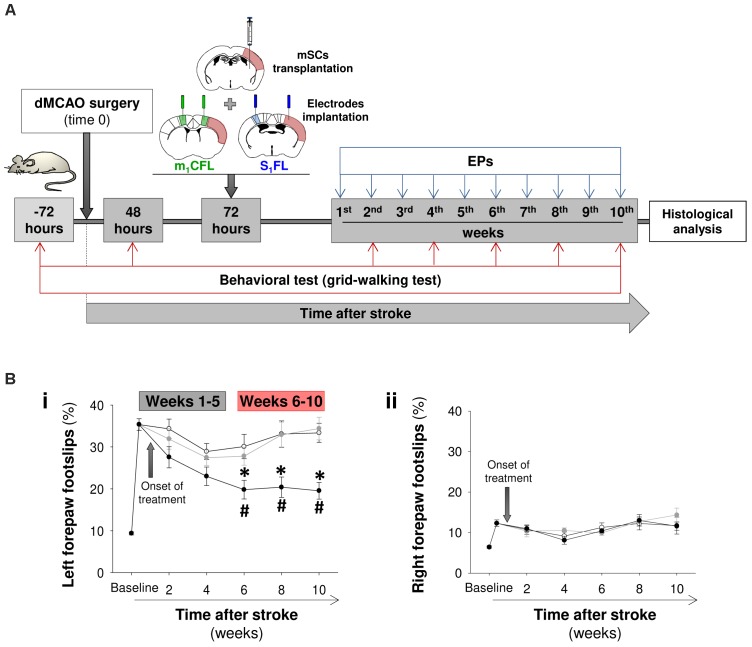
Sensorimotor recovery in stroke mice treated with mSCs-silk fibroin hydrogels. **(A)** Schematic of the study performed to examine the temporal progression of sensorimotor function and evoked activity in FLs_1_ and cFLm_1_ regions. The stroke animals were divided into three groups and treated with a saline solution (PBS, white circles in **B**), mSCs (gray circles) or mSCs encapsulated in silk fibroin hydrogels (black circles). Sensorimotor coordination was evaluated with the grid-walking test before and after stroke at different post-treatment time points. The FLs_1_ and cFLm_1_ responses were obtained for several weeks after treatment. The schematics of coronal brain slices illustrate the site of cerebral injection (treatment) and the placement of electrodes in relation to the cortical damage (soft red) induced in this specific mouse stroke model. **(B)** Percentages of footslips examined with the grid-walking test for the left (panel **i**) and right forepaws (panel **ii**), respectively. The left forepaw was contralateral to the infarcted (right) hemisphere. Data are presented as the means ± the SEM from nine mice per group at the onset of the study (PBS, mSCs or mSCs-Silk fibroin). Asterisks denote significant differences in data at every time point analyzed between the mSCs-SF group and the other two treated groups. The hashed symbols show statistically significant differences compared with the baseline post-stroke pretreatment values (two-way ANOVA followed by Tukey’s test; ^∗^*p* < 0.05).

In a new study, different groups of mice were cerebrally injected with a saline solution (PBS), mSCs in PBS solution or mSCs embedded into SF. Prior to the transplant, mSCs were expanded *ex vivo* and functionally characterized (**Supplementary Figure S2**). On same day, coinciding with 72 h post-stroke, two sequential surgeries were performed to inject the cells and implant cortical electrodes (**Figure 2A**). In the first 48 h after stroke (before treatment), the percentage of footslips with the forelimb contralateral to the infarcted hemisphere was dramatically increased compared with the pre-stroke (baseline) condition (**Figure 2B**). No evidence of functional recovery was observed in mice injected with PBS or mSCs in PBS over time after treatment. However, gradual sensorimotor recovery was observed in the mice treated with mSCs-SF. Considering the temporal evolution of the mSCs-SF group, we did not observe statistically significant differences in the first weeks post-treatment compared with the other two animal groups or with the pretreatment condition, but we did observed significant differences in the interval from 6 to 10 weeks.

**FIGURE 3:**
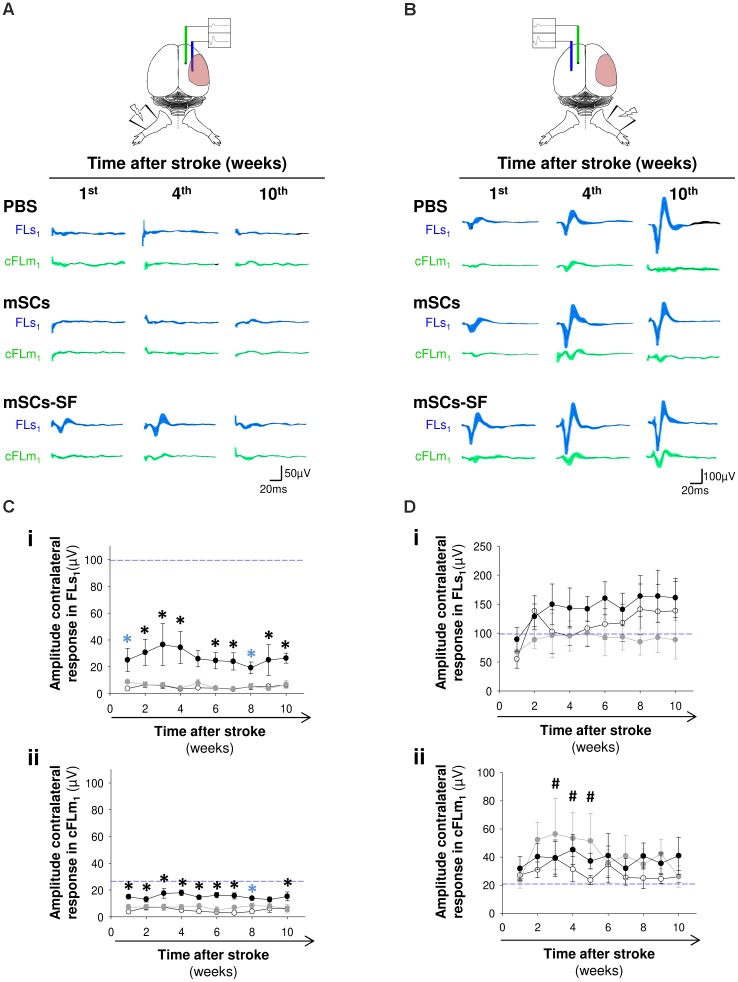
Evoked cortical activity in the infarcted and non-infarcted hemispheres of stroke mice after treatment with mSCs and silk fibroin polymers. **(A)** Average recordings of evoked activity in FLs_1_ and cFLm_1_ areas of the infarcted hemisphere in the group of mice treated with PBS, mSCs or mSCs-SF at discrete time points after treatment. **(B)** Mean recordings of cortical activity obtained for the non-infarcted hemisphere. **(C,D)** Amplitude of evoked activity in FLs_1_ and cFLm_1_ in the infarcted **(C)** and non-infarcted **(D)** hemispheres of mice injected with PBS (white circles), mSCs (gray circles) and mSCs encapsulated in silk fibroin (black circles) over time after treatment. In all graphs, the dashed blue lines represent the average activity in FLs_1_ (panels **i**) and cFLm_1_ (panels **ii**) under non-stroke condition. Data are presented as the means ± SEM from nine mice per group at the onset of the study. For every time point analyzed, the black asterisks denote significant differences between mSCs-SF mice and the other two studied groups, PBS and mSCs. Blue asterisks show statistically significant differences between PBS and mSCs-SF groups (two-way ANOVA followed by Tukey’s test; ^∗^*p* < 0.05). The hashed symbols denote significant differences (^#^*p* < 0.05) between the cFLm_1_ responses in mSCs mice compared to the pre-stroke condition (dashed blue line).

The lack of sensorimotor recovery in PBS and mSCs groups paralleled the lack of evoked activity in FLs_1_ and cFLm_1_ of the infarcted hemisphere (**Figures 3A,C**), denoting a substantial injury of the forepaw sensory representation (FLs_1_), as was initially characterized in this stroke model (**Figure 1**). However, in the damaged hemisphere of the mSCs-SF group, a residual but still significant evoked activity was detected in both cortical representations from the first week post-treatment and during the time period analyzed (**Figures 3A,C**). In contrast, when we considered the non-infarcted hemisphere, clear evoked activity was observed in all studied groups (**Figures 3B,D**). We also examined the interval of latencies between the maximal responses detected in FLs_1_ and cFLm_1_ in both hemispheres, finding that in the mSCs-SF group the latency value in the infarcted hemisphere was reduced (at least in weeks 6–7 post-treatment) compared with the average latency estimated for the same hemisphere in non-infarcted mice (**Supplementary Figure S3A**).

**FIGURE 4:**
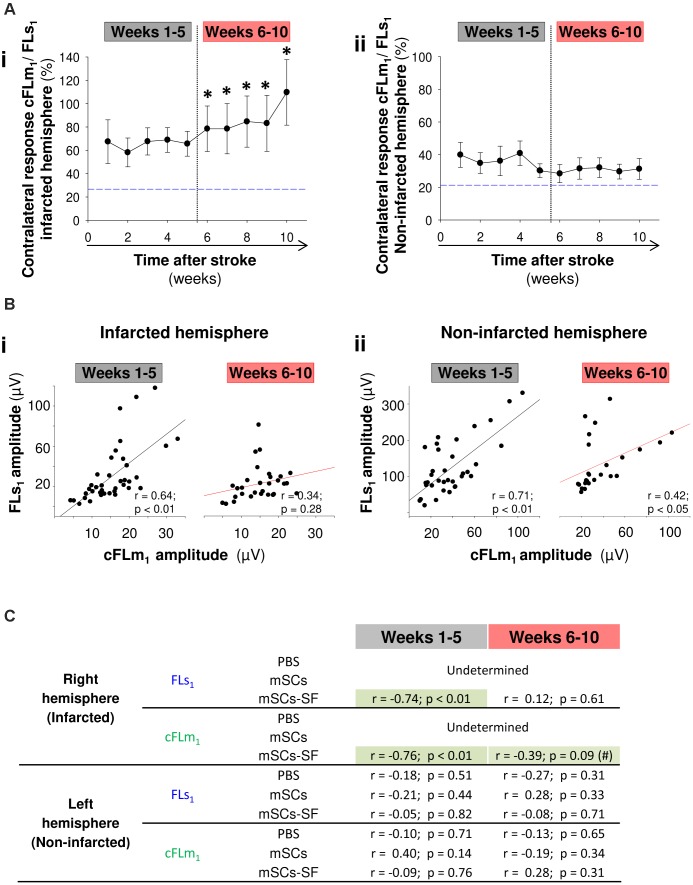
Loss of connectivity between FLs_1_ and cFLm_1_ representations underlies the functional recovery in stroke mice treated with mSCs and silk fibroin polymers. **(A)** cFLm_1_/FLs_1_ ratio in the infarcted (panel **i**) and non-infarcted (panel **ii**) hemispheres. The weeks corresponding with both periods of sensorimotor recovery (examined with the grid-walking test) are shown in both panels. The dashed blue lines represent the cFLm_1_/FLs_1_ ratio for both hemispheres under the non-stroke condition. **(B)** Pearson’s correlation coefficients for the maximal amplitude of responses in FLs_1_ and cFLm_1_ from the infarcted (panel **i**) and non-infarcted (panel **ii**) hemispheres in both intervals of recovery (weeks 1–5 and weeks 6–10). **(C)** Pearson’s correlation coefficients for the sensorimotor deficits examined with the grid-walking test and the maximal amplitude of responses in FLs_1_ and cFLm_1_ from the non-infarcted (left) and the infarcted (right) hemispheres for all treated mice included in the present this study. The black asterisks in the data obtained from mSCs-SF-treated mice denote significant differences between the post-stroke FLs_1_/cFLm_1_ ratio and the values obtained under the non-stroke condition (blue dashed line; one-way ANOVA; ^∗^*p* < 0.05).

An interesting finding with the mSCs-SF group was the temporal pattern of the cFLm_1_/FLs_1_ ratio in the infarcted hemisphere, which exhibited a biphasic evolution (**Figure 4A**). In the first few weeks, when non-significant recovery was observed on the grid-walking test (**Figure 2B**), the cFLm_1_/FLs_1_ ratio remained relatively constant and did not show significant differences compared to the pre-stroke (blue dashed line) condition (**Figure 4A**). However, a progressive and significant increase in the cFLm_1_/FLs_1_ ratio was observed in the last few weeks after treatment (**Figure 4A**), the precise time at which significant sensorimotor recovery was observed in the mSCs-SF mice (**Figure 2B**). The increase in the cFLm_1_/FLs_1_ ratio resulted from a net decrease in FLs_1_ activity and an increase in the responsiveness of cFLm_1_ (**Supplementary Figure S3B**). However, no significant variation in the cFLm_1_/FLs_1_ ratio was observed in the non-infarcted hemisphere of mSCs-SF mice, with values similar to the pre-stroke condition (**Figure 4A**).

In the mSCs-SF mice, the association of a progressive increase in the cFLm_1_/FLs_1_ ratio (infarcted hemisphere) with increased cFLm_1_ activity and decreased FLs_1_ responsiveness suggests an impairment of functional connectivity between FLs_1_ and cFLm_1_ when gradual sensorimotor recovery occurs. This assertion is based on the following facts. First, when we exclusively consider the non-damaged hemisphere, similar to the pre-stroke condition, a positive correlation between cFLm_1_ and FLs_1_ responses was determined independently of the two periods of sensorimotor dexterity (**Figure 4B**). Furthermore, in this hemisphere we did not identify any correlation between the maximal responses recorded in FLs_1_ and cFLm_1_ with the footslips of the affected paw, which was located ipsilateral to this hemisphere (**Figure 4C**), suggesting that the level of FLs_1_ and cFLm_1_ activity in the non-infarcted hemisphere had no effect on the evolution of sensorimotor recovery in mSCs-SF mice. Second, in the infarcted hemisphere, the maximal activation recorded in FLs_1_ correlated with the maximal cFLm_1_ response during the first weeks but not during the period of significant sensorimotor recovery (**Figure 4B**). During the first weeks, the footslips by the affected paw exhibited equivalent correlations with the responses recorded in both FLs_1_ and cFLm_1_ (**Figure 4C**); however, during weeks 6–10, only the cFLm_1_ responses displayed a moderate correlation with the sensorimotor deficits (*p* = 0.09; **Figure 4C**). Finally, both responses (FLs_1_ and cFLm_1_) in the non-infarcted hemisphere of PBS- or mSCs-treated animals positively correlated throughout study period (**Supplementary Figure S4**), suggesting that this unilateral infarction model did not impair the functional connectivity between both cortical maps in this hemisphere in any of the three studied groups.

In summary, the significant recovery observed in mSCs-SF treated mice several weeks after treatment (weeks 6–10) was apparently associated with a lack of functional connectivity between FLs_1_ and cFLm_1_ in the damaged hemisphere (**Figure 6**). In this late period, the activity generated in cFLm_1_ in response to forelimb stimulation no longer depended on the previous response in FLs_1_, indicating that cFLm_1_ probably represented the sensory afferent information of the forelimb (**Figure 6**). Under this condition, the activity recorded in cFLm_1_ moderately correlated with the degree of sensorimotor dysfunction, suggesting that the emerging sensory activity in cFLm_1_ was in part responsible for the recovery of sensorimotor coordination and function.

### Neuroprotection Supports the Cortical Remodeling Underlying Functional Recovery

**FIGURE 5:**
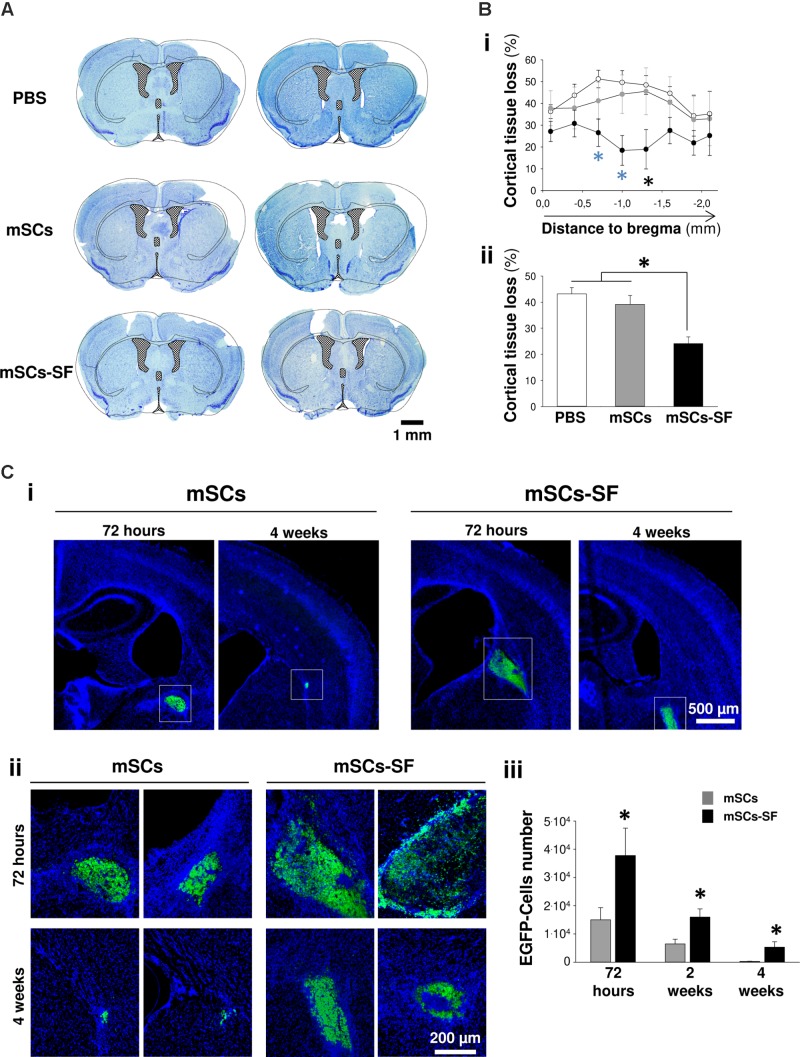
Increased survival of transplanted mSCs engrafted into silk fibroin hydrogels enhances neuroprotective effects after stroke. **(A)** Representative images of Nissl-stained coronal brain sections performed 10 weeks after injection of PBS, mSCs alone or mSCs encapsulated in silk fibroin hydrogels (images showed from two different mice in each group). Note how the majority of the cortical territory was preserved in stroke mice co-injected with mSCs and silk fibroin gels. **(B)** Panel **i** shows the regional distribution of the cortical tissue loss (damaged tissue) in coronal sections along the rostrocaudal axis as measured from bregma. Panel **ii** shows the total cortical tissue loss. **(C)** Representative images at low (panel **i**) and high (panel **ii**) magnification of mSCs expressing the EGF protein (EGFP) implanted into the striatum of non-EGFP-expressing mice, 72 h and 4 weeks after the injection of mSCs or mCSs-SF. Every image in panel **ii** corresponds to sections examined from different mice. In the figure, nuclei were stained with Hoechst dye (pseudocolored blue). Panel **iii** shows the quantification of EGFP-positive cells at different time points after transplantation. The asterisks in (**B**, panel **i**) show significant differences between mice treated with PBS or mSCs-SF (blue asterisks) or between mSCS-SF animals compared with the other two mice groups analyzed (black asterisk) (two-way ANOVA followed by Tukey’s test; ^∗^*p* < 0.05). The asterisks in (**B**, panel **ii**) show significant differences between groups (one-way ANOVA test followed by Tukey’s test; ^∗^*p* < 0.05). The asterisks in C (panel **iii**) denote statistically significant differences between groups at each time point analyzed (two-way ANOVA followed by Tukey’s test; ^∗^*p* < 0.05).

In our study, an intriguing question was related to the mechanisms underlying both: (i) the residual somatosensory activity in FLs_1_ that was detectable from the first days after implantation of mSCs-SF (**Figure 3A**) and (ii) the delayed (6–10 weeks post-treatment) emergent sensory activity in peri-infarcted areas within the cortical cFLm_1_ representation (**Figure 4A**). In order to assess these questions the loss of cortical mass after stroke as a function of the treatment was analyzed. It was found that ten weeks after treatment, the mice that had been previously injected with PBS or mSCs in PBS showed a strong loss of cortical tissue in the infarcted hemisphere compared with animals implanted with mSCs-SF (**Figures 5A,B**). In another set of experiments, a strong cortical loss after stroke was detected as early as 96 h after treatment, even in mice that were only injected with SF, whereas cortical damage was only significantly reduced at this time point in the group of mice implanted with mSCs encapsulated into the biomaterial (**Supplementary Figure S5**). The significant neuroprotective effect was favored by the inclusion of this cell population in SF, since the injection of mSCs alone did not offer a significant advantage in reducing the extent of brain damage compared with the animals that had merely been injected with PBS. We also inferred that the biomaterial exerted a potential benefit based on the results from alternative studies of intracerebral implantation of mSCs (EGFP-positive) into non-EGFP mice, where a large number of mSCs were engrafted for longer periods when this cell population was integrated into SF hydrogels (**Figure 5C** and **Supplementary Figure S6**).

**FIGURE 6:**
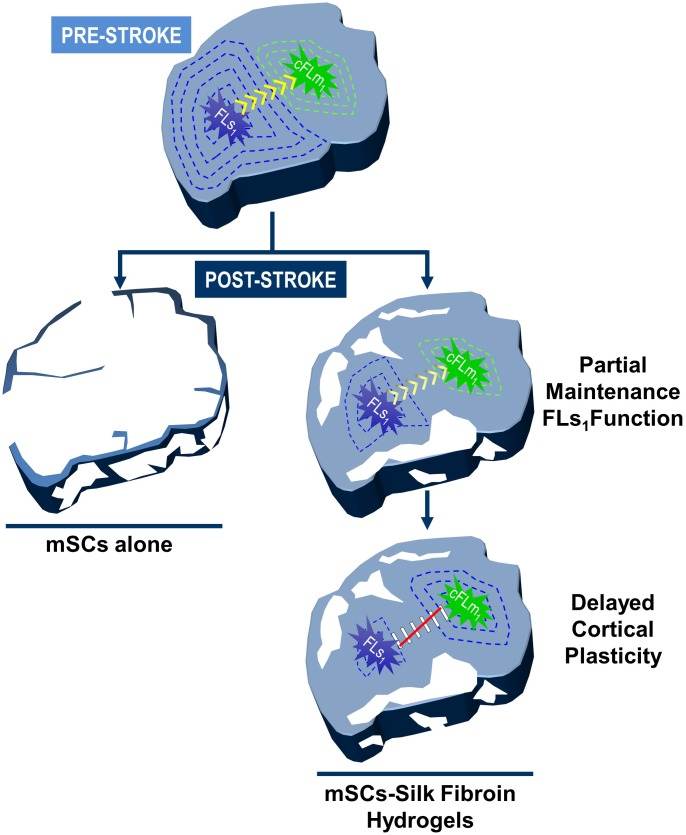
Cortical remodeling and the emergence sensory activity in cFLm_1_ underlies post-stroke functional recovery after transplantation of mSCs encapsulated in silk fibroin hydrogels. Model showing the two steps of mSCs-SF-based therapeutic action determined in the present study: neuroprotection and cortical reorganization. The top panel shows the somatotopic areas corresponding to the forepaw somatosensory (FLs_1_) and motor (cFLm_1_) representations. Under the pre-stroke condition, a cortical response in FLs_1_ emerges after forelimb stimulation (blue concentric dashed lines), which subsequently is transmitted to cFLm_1_ (green concentric dashed lines) indicating the dependency of both cortical maps on a functional circuit (yellow arrows). After stroke, the transplantation of mSCs encapsulated in silk fibroin hydrogels decreased the area of the affected cortical territory (white gaps represent the damaged loss tissue), partially preserving the evoked activity of both cortical representations in response to forelimb stimulation (illustrated as a reduction in the number of concentric blue and green lines). Several weeks after treatment, coinciding with the phase of significant sensorimotor recovery, a functional disconnection was produced between both cortical maps (red line). In this stage, the cFLm_1_ area represented the sensory information after forelimb stimulation (transition from green to blue lines in cFLm_1_ area), whereas a gradual loss of responsiveness was observed in the FLs_1_ map. In contrast, the stroke animals injected with mSCs alone or PBS showed a significant loss of cortical territory and complete abolition of activity in both cortical maps that was translated into profound and permanent sensorimotor behavioral deficits and recovery was not observed in the time period examined (left panel of the figure).

## Discussion

For most patients, effective therapies for repairing the damaged brain are not currently available. Conceptually, behavioral improvement might be related to (i) cortical reorganization of peri-infarcted regions in the affected (Brown et al., 2009) and non-affected (Dijkhuizen et al., 2001) hemispheres; (ii) the reestablishment/restitution of circuitry in the damaged tissue (Taguchi et al., 2004; Ramos-Cabrer et al., 2010; Wang et al., 2016); and (iii) compensatory postural strategies that are particularly relevant in patients with large cerebral infarcts involving the motor cortex (Farr and Whishaw, 2002; Knieling et al., 2009; Moon et al., 2009). Regarding the first mechanism, in most cases, researchers have not been able to define whether cortical reorganization is maladaptive (Kim et al., 2015), otherwise unrelated (Xerri et al., 1998) or participates (Castro-Alamancos and Borrel, 1995; Nudo et al., 1996b; Dijkhuizen et al., 2001; Shanina et al., 2006; Andres et al., 2011) in the temporal pattern of functional recovery, making the establishment of appropriate intervention programs to drive efficient cortical plasticity difficult.

In our study, we did not detect significant signs of recovery in the stroke animals that had been acutely injected with PBS or mSCs alone; these animals showed permanent sensorimotor deficits (examined with the grid walking test) and complete lack of evoked activity in the somatosensory and motor cortices. The lack of evoked responses is mostly compatible with extensive damage to the cortical territory, which might justify the poor functional outcomes characteristic of large infarctions (Whishaw, 2000; Moon et al., 2009). In contrast, progressive and significant recovery was exclusively observed in mice implanted with mSCs encapsulated in SF hydrogels. Two sequential phenomena might tentatively explain the positive effect of this therapy on this group of animals. First, the reduced cortical damage detected after treatment probably ascribed to the well-known neuroprotective properties of mSCs (Steinbeck and Studer, 2015; Trounson and McDonald, 2015), properties that were visibly potentiated when this cell population was implanted together the biomaterial. The lower injury on the cortex was translated into a partial preservation of evoked responses in FLs_1_ and cFLm_1_ areas and reduced sensorimotor deficits from the first weeks after treatment. Second, and probably more relevant for recovery, the lower amount of cortical damage in the mSCs-SF animals might constitute a prerequisite to promote delayed cortical plasticity in the tissue surrounding the lesion, leading to behavioral improvements. Current evidence favors the existence of reorganization of the peri-infarct areas in infarctions covering small regions of cortical territory (Gonzalez and Kolb, 2003; Dancause et al., 2006; Murphy and Corbett, 2009). This finding is consistent with the lack of emerging sensory activity in cFLm_1_ or any evidence of plasticity examined under our experimental conditions in the animals injected with PBS or mSCs alone, which showed large damage in the sensorimotor cortex and poor recovery, as described above. Although the reorganization of cortical maps in the peri-lesional tissues in most studies with human and animals have been examined with intracortical stimulation or transcranial magnetic stimulation (humans), remodeling events were inferred in our study from the recordings of evoked cortical activity after forelimb stimulation, recapitulating the peri-lesional reorganization previously described in animals that exhibited spontaneously recovery or recovered after physical rehabilitation (Nudo et al., 1996b; Dancause, 2006; Brown et al., 2009).

Different engineering approaches are being used to increase stem cell survival and potentiate the neuro-secretome ability of different stem cells. Genetic engineering, pre-conditioning and cell encapsulation into biomaterial scaffolds constitute technological opportunities in the field (Bernstock et al., 2017). Particularly interesting is the ability of several biomaterials to enhance the survival, retention and integration of therapeutic cells in the brain tissue (González-Nieto et al., 2018). This is the case for hyaluronic acid (Ballios et al., 2015; Moshayedi et al., 2016), PLGA (Bible et al., 2009), matrigel (Jin et al., 2010), collagen (Yu et al., 2010), hyaluronan-methylcellulose (Ballios et al., 2015) or thermoreversible polymers (Osanai et al., 2010) among others. This nervous tissue-biomaterial-cells triad has been preferably exercised with hydrogel-based biomaterials, which show unique mechanical properties in the range of soft tissues like the nervous tissue and therefore might mimic with certain similarity the *in vivo* microenvironment. Because the list of biomaterials for brain repair is rapidly expanding there is an important need to identify the more suitable materials in terms of efficacy and compatibility. In our study, we tested for first time the efficacy of combining mSCs with SF to repair the stroke-damaged brain. Among the spectrum of possible biomaterials, SF is a flexible and adaptable natural polymer that has been used for years in many clinical applications including surgical sutures, mechanical support for cruciate ligament or reconstructive and aesthetic surgery. Its null immunogenic response, non-toxic properties and inertness respect other biomaterials makes it a strong biomaterial candidate for tissue regeneration. In addition this biomaterial shows good compatibility and tolerability with central nervous system (Fernandez-Garcia et al., 2016). Alternative engineering materials under development have strong potential but also are subjected to certain limitations. For example, hyaluronic acid (HA), a very common biomaterial used in experimental stroke (Ballios et al., 2015; Nih et al., 2018) might exerts different effects depending of the size of the polymer. The fragments generated after degradation might trigger immuno-reactivity and inflammatory events (Petrey and de la Motte, 2014). Furthermore, the accumulation of HA has been associated with aging and demyelinating disorders such as multiple sclerosis (Back et al., 2005) and HA might be detrimental for remyelination of neural circuits. Biomaterials like chitosan or PLGA have been associated with inflammation and allergic reactions; matrigel, a mix of extracellular matrix proteins from mouse sarcoma is not suitable for human subjects. Collagen shows important safety problems in relation to the possible contamination with viruses and prions and degrades rapidly.

In comparison with the implantation of mSCs alone a greater content of EGFP-mSCs was observed in the brain after transplantation with SF hydrogels. Despite their known immunosuppressive and anti-inflammatory properties it has been extensively reported that the engraftment of mSCs is quite limited in the host brain (Munoz et al., 2005; Coyne et al., 2006, 2007; Ohtaki et al., 2008; Moloney et al., 2010). A strong reduction of mSCs survival was found in hippocampus and striatum as soon as 1 week after implantation (Munoz et al., 2005; Ohtaki et al., 2008; Moloney et al., 2010). Rapid rejection of mSCs has been attributed to inflammatory responses mostly mediated by microglia and macrophages although these mechanisms are not completely elucidate. In our study, the EGFP transgene might tentatively contribute to this detrimental effect since a cytotoxic T lymphocyte response against the EGFP antigen cells has been detected after transplantation of EGFP-lymphoma cells in non-EGFP immunocompetent syngeneic mice (Stripecke et al., 1999) and treatment with cyclosporine A enhances the survival of EGFP-mSCs implanted in the striatum and the subarachnoideal space (Cobacho et al., 2009; Moloney et al., 2010). However, GFP/EGFP reporters has been commonly used to track axonal projections and neuronal connectivity for example after cerebral implantation of GFP expressing embryonic stem cells into non-EGFP mouse brains (Gaillard et al., 2007; Michelsen et al., 2015) and in the absence of cyclosporine A no differences in survival were observed between non-EGFP- and EGFP-mSCs implanted into the brain striatum (Moloney et al., 2010). In any case, the biomaterial used in the present study might plausibly constitute a structural scaffold to partially protect mSCs from a possible inflammatory response avoiding their rapid rejection by the host brain.

Because a main objective of our study was to establish the mechanistic aspects of recovery at functional level (connectivity between somatotopic maps), a limitation of our study relates to the analysis of the cellular/molecular mechanisms behind the neuroprotective ability of mSCs encapsulated into SF. The neuroprotection exerted by mSCs-SF might be inferred from the reduction of the damage area after the occlusion of the MCA and from the partial preservation of evoked cortical activity from the first week post-implantation. The therapeutic action of mSCs embedded into SF might be due to a synergic effect involving a higher long-term survival of mSCs in the host tissue and modifications of their secretome function. Recent *in vitro* results from our group (in preparation) indicates that this biomaterial reduces the secretion of BDNF, SDF1-Alpha and VEGF from mSCs, while substantially augments (up to 10 times more) the secretion of TGF-Beta1, a known anti-inflammatory and angiogenic factor that is strongly induced after brain injury polarizing microglia to an anti-inflammatory phenotype (Rustenhoven et al., 2016) increasing functional recovery after acute brain injury (Taylor et al., 2017). In relation to the angiogenic potential of TGF-Beta1, brain plasticity and post-stroke neurological recovery have been related with increasing angiogenesis (Chen et al., 2003). In this context it is also interesting the strong ability of SF to support endothelial cell survival and growth as well as the formation of microvascular networks *in vitro* and *in vivo* (Unger et al., 2007; Stoppato et al., 2013).

By contrast, a controversial aspect of our study relies in the lack of any post-stroke therapeutic effect ascribed to the mSCs implanted without the biomaterial in relation to the injection of PBS. Numerous studies have reported favorable outcomes after transplantation of mSCs (Vu et al., 2014). However, the effects of mSCs are largely variable depending of administration route, temporal window of administration, cell dosage and tissue source, animal model and type of stroke. In alternative studies no functional recovery has been observed after treatment with mSCs (Qu et al., 2009; Scheibe et al., 2012; Quittet et al., 2015). Discrepancies between positive and negative results are not yet clarified, although application route, cell dosage and final read outs employed to examine functional outcomes might contribute. Across the literature the highest therapeutic effects of mSCs were observed when this cell population was implanted in the early hours after stroke, typically 24 h, in our case 72 h. Another possible variable might be related with the area of brain implantation, striatum in our study in comparison with other brain regions (stroke cavity, peri-lesional cortex in cortical stroke models). We believe that a main factor is the mouse strain used in this study, CD-1. In contrast to the more popular mouse strain (C57Bl6) used in the majority of studies, in our work we choose this strain (CD-1) because in our hands the infarction size and the functional deficits (examined by behavioral testing and electrophysiology) were more consistent and reproducible than in C57Bl6 (Barios et al., 2016). Infarction volumes in CD-1 are generally higher because of poor collateral vasculature and higher susceptibility to hypoxia. Thus, our election was to test the efficacy of this experimental treatment in a very restricted condition, which could have repercussion in the ability of mSCs to exert a significant response. The cortical tissue loss tended to be smaller after implantation of mSCs alone although this effect was not statistically significant (**Supplementary Figure S5**). Under this condition we found a complete abolition of cortical evoked response that contrasted with the partial preservation (from the first week post-treatment) of cortical function in mice treated with mSCs and SF together.

In humans and in a majority of stroke models, the reorganization of cortical maps has mainly been observed between motor representations surrounding the damage area (Dancause, 2006). In stroke rodent models affecting the somatosensory forepaw area, a delayed emerging sensory activity (several weeks after stroke) was observed in peri-lesional areas corresponding to the motor cortex, coinciding with the temporal pattern of spontaneous sensorimotor recovery (Brown et al., 2009; Harrison et al., 2013). In our case, the fundamental mechanisms by which cFLm_1_ regulates direct sensory processing and whether the canonic function of the remaining (non-affected) motor cortical map is still preserved or also extended to other nearby motor areas under this condition are not known. A probable first requirement for this vicarious process is associated with the reciprocal functions between cortical maps that share similar elements of neuronal network architecture, regardless of whether they are involved in direct sensory or motor processing. Considering the functional organization of the barrel cortex as an example, the somatosensory barrel representation might physiologically contribute to motor control of whisker retraction in rodents (Matyas et al., 2010). Thus, the promiscuity of cFLm_1_ for sensorial and motor processing may not be surprising if somatosensory and motor areas are functionally interchangeable (Brown et al., 2009; Harrison et al., 2013). However, some exceptions to this “rule” have been reported, since infarctions restricted to the motor cortex do not produce substantial reorganization of sensory representations (Harrison et al., 2013). Several possibilities have been proposed to explain the emerging sensory activity in peri-lesional cFLm_1_ areas, such as the unmasking of somatosensory circuitry (Jacobs and Donoghue, 1991). Alterations in GABAergic inhibitory neurotransmission have been proposed to be primarily responsible for the somatotopic reorganization to define cortical territories (Foeller et al., 2005; Hensch, 2005). Inhibition of GABAergic signaling restricted to the forelimb motor cortex causes fast reorganization (in the range of minutes) of cortical maps in adult rats, which showed evoked forelimb movement when the vibrissa representation was electrically stimulated (Jacobs and Donoghue, 1991). Moreover, the inhibition of GABAergic signaling favors the rearrangement of neural networks in the peri-infarct region supporting functional recovery. For example, when a α5-GABA_A_ antagonist was chronically applied beginning on day 3 after stroke, functional recovery was observed as early as 7 days after ischemia (Clarkson et al., 2010), a time period observed in other studies of therapeutic post-stroke GABAergic signaling inhibition (Alia et al., 2016). Early inhibition of GABAergic signaling appears to accelerate functional recovery compared with the findings from alternative studies showing spontaneous recovery (Brown et al., 2009) or a neural stem cells transplantation-induced (Andres et al., 2011) delay in functional recovery, which similar to our study, were characterized by a significant sensorimotor improvement weeks after stroke. Different structural changes (synaptogenesis, dendritic length and branching, axonal sprouting, dendritic spine turnover) might also explain the delayed emergence of sensory activity in cFLm_1_ (Stroemer et al., 1993, 1995; Carmichael et al., 2001; Gonzalez and Kolb, 2003; Brown et al., 2007, 2008, 2009; Li et al., 2010, 2015; Andres et al., 2011; Overman et al., 2012). In addition, mSCs might be involved in the paracrine release of diverse factors that stimulate axonal and dendritic plasticity as well as synaptogenesis (Li et al., 2006; Liu et al., 2008, 2010; van Velthoven et al., 2012). The structural rearrangement in the peri-lesional cortex might be favored by structural modifications in pre-existing neuronal/glia networks, as well as the functional integration of newborn neurons that facilitate functional plasticity (Shiromoto et al., 2017) due to the secretory activity of mSCs (Munoz et al., 2005).

In a recent study, delayed post-stroke recovery occurred after the combined transplantation of neural stem cells and progenitors (nSCs) and the 3K3A-APC protease to enhance the survival and proliferation of the cerebrally transplanted cells (Wang et al., 2016). Behavioral improvements resulted from the functional integration of nSCs-derived neurons in the territory affected by the infarction in parallel with cortical hyperplasia (Wang et al., 2016). In another example, a delay in the functional restoration of the damaged representation was also observed after treatment with a C17.2 stem cell line, but no evidence of brain reorganization was detected in peri-lesional areas (Ramos-Cabrer et al., 2010). In any case, if endogenous neuronal integration supported the delayed recovery of our treated animals, it would functionally occur through modifications in the perilesional cortex, but not in the damaged forepaw sensory area (FLS_1_), since a gradual, unexplained loss of activity, was observed in the FLS_1_ map over time after stroke, reaching values of residual activity that did not correlate with sensorimotor abilities during the significant recovery phase.

We are still far from obtaining a comprehensive picture of the molecular and cellular substrates underlying cortical reorganization in the peri-lesional cortex and how these mechanisms are cleverly orchestrated to restore damaged functions in response to post-stroke signaling. In this study, we have revealed at functional level how the infarcted brain might recover from injury after the combined transplantation of stem cells and SF hydrogels. We report for the first time to our knowledge, that cortical maps can be reshaped after an experimental paradigm based on the transplantation of stem cells of mesenchymal origin, whose neuroprotective function was potentiated by this innocuous polymer, confirming the suitability of this biomaterial in this specific application. Our findings contribute to the rational search for therapies designed to enhance cortical plasticity and recovery after cerebral damage, expanding the list of revolutionary biomaterials that serve as a support for the engraftment and function of stem cells with neuro-therapeutic abilities. Finally, our results may pave the way for studies aiming to understand how specific types of peri-lesional plasticity contribute to functional recovery.

## Author Contributions

GG and DG-N conceived the idea and supervised the whole project. LF-G performed the majority of the experiments with the help of JP-R, MR, RM-M, FP, and DG-N. LF-G and DG-N analyzed the data. DG-N interpreted the data and wrote the paper.

## Conflict of Interest Statement

The authors declare that the research was conducted in the absence of any commercial or financial relationships that could be construed as a potential conflict of interest.
