# Supplementary material for: Cortical Reshaping and Functional Recovery Induced by Silk Fibroin Hydrogels-Encapsulated Stem Cells Implanted in Stroke Animals
Source: Front Cell Neurosci. 2018 Sep 6;12:296. doi: 10.3389/fncel.2018.00296 (PMC6135908; doi:10.3389/fncel.2018.00296)
Supplement: Supplementary file 1 [file Data_Sheet_1.doc]

**SUPPLEMENTARY MATERIAL**

**Cortical Reshaping and Functional Recovery Induced by Silk Fibroin Hydrogels-Encapsulated Stem Cells Implanted in Stroke Animals**

Laura Fernández-García, José Pérez-Rigueiro, Ricardo Martínez-Murillo, Fivos Panetsos, Milagros Ramos, Gustavo V. Guinea, and Daniel González-Nieto

Supplementary material consists of:

1. Supplementary Methods

1. Supplementary Figures:

♦ Supplementary Fig. 1- Characterization of damage brain extension and sensorimotor evaluation after MCA occlusion (stroke model)

♦Supplementary Fig. 2- Functional characterization of bone marrow mesenchymal stem stromal cells

♦Supplementary Fig. 3- Latency interval of responses between FLs_1_ and cFLm_1_.Temporal evolution of contralateral responses in FLs_1_ and cFLm_1_ at 2 and 10 weeks post-treatment

♦Supplementary Fig. 4- Correlation studies of intracortical connectivity between FLs_1_ and cFLm_1_ of the infarcted and non-infarcted hemispheres in all treated mice groups

♦Supplementary Fig. 5- Cortical tissue loss and subcortical expansion at short and long time after treatment

♦Supplementary Fig. 6- Temporal and spatial dynamics of EGFP-expressing mSCs implanted in the mouse brain

**1. Supplementary Methods**

*Isolation and Preparation of Cells*

Bone marrow (BM) mesenchymal-multipotent stromal cells (BMmSCs) were isolated from donor adult CD1-wild type or C57BL/6-Tg(CAG-EGFP)C14-Y01-FM131Osb mice. Fresh BM was extracted and processed as previously described . Briefly, after cervical dislocation mouse BM was isolated from femora, tibiae and pelvis. The bones were crushed in a mortar with cool PBS (Gibco® life tecnologies) to obtain a homogeneous cell suspension, which was sieved through a 70 μm-cell strainer, centrifuged (5 min at 1.500 rpm) and incubated 5 min at room temperature in lysis solution (BD Pharm Lyse™) to discard the red blood cells. BM cells were plated at density of 6.5 × 10^5^ cells/cm^2^, on fibronectin-coated flaks (4µg/cm2; Sigma aldrich; Cat# F4759) in Iscove Modified Dulbecco Medium (IMDM) supplemented with 20% of mesenchymal serum (Stem Cell Technologies, Cat#05502), 100 U/ml penicillin/0.1 mg/ml streptomycin (gibco® life technologies), 100μM 2-mercaptoethanol (Fisher Scientific), 2mM L-glutamine (gibco® life tecnologies), 10 ng/ml human platelet-derived growth-factor (hPDGF-BB; Peprotech®; Cat# 100-14B), and 10 ng/ml recombinant mouse epidermal growth factor (rmEGF; Peprotech®; Cat# 315-09). Cells were incubated at 37ºC in 5% CO2 for 72 hours. At this time, the non-adherent cells were removed replacing half of the medium. Hemi-depletion was performed three times every week.

*Characterization of mSCs*

Immunophenotypic analysis of BM cells and mSCs was performed after immunolabeling with rat antibodies against mouse-CD45 (BD; Cat# 559864), mouse-CD31 (BD; Cat# 551262) and mouse-Ter119 (BD; Cat# 17-5921), all antibodies conjugated with allophycocyanin (APC). A Rat IgG2a anti-mouse antibody APC-conjugated (BD; Cat# 553932) was used as isotype control. BM mSCs were defined as CD45^Neg^ Ter119^Neg^CD31^Neg^. Flow cytometry acquisition was carried out in a BD FACSCanto II system (Becton Dickinson, California, USA). At least 1x10^5^ events were acquired from each sample (in triplicate) on the APC channel (excitation at 633 nm and emission at 660 nm). Gating and analysis of events was accomplished with FlowJo software package (FLOWJO, LLC, Oregon, USA). Thresholds were set at ~99% of negative population for the isotype antibody control and maintained throughout the analysis. Cell acquisition was performed through Hospital Ramón & Cajal Core Facility for Flow Cytometry (Madrid, Spain). The multipotent differentiation of mSCs was assessed as previously described with some modifications . Conditioned culture media containing IMDM with 20% FBS, 100 U/ml penicillin, 0.1 mg/ml streptomycin, 100μM 2-mercaptoethanol, 2mM L-glutamine and 0.1μM dexamethasone (FisherScientific) was supplemented with 0.25mM ascorbic acid (Sigma Aldrich; Cat# A5960), and 10mM 2-glycerolphosphate (Sigma Aldrich; Cat# G6501) for osteoblast diiferentiation; with 100µM indomethacin (Sigma Aldrich; Cat# I7378) and 5µg/ml insulin (Sigma Aldrich; Cat# I9278) for adipocyte commitment; or with 10ng/ml TGF-β1 (Peprotech®; Cat# 100-21) for chondrocyte differentiation. Cell cultures were incubated at 37º C, 5% CO2 and 100% humidity for approximately 3-4 weeks. Osteoblasts, adipocytes and chondrocytes were identified by positive staining for alkaline phosphatase (Sigma-Aldrich; Cat# 86R), oil red (Acros organics; Cat# 189004250) and toluidine blue (Sigma Aldrich; Cat# T3260) respectively.

*In Vitro Survival of mSCs Enclosed into Silk Fibroin Hydrogels*

In a set of experiments 1x10^5^ mSCs were mixed with 500 µl of sonicated SF solution in pre-gel state. After 72 hours or two weeks in culture, 1 µg/µl of the vital marker Calcein-AM (eBioscience; Cat# 65-0853) was added to the culture for labeling of mSCs encapsulated in SF hydrogels, The identification of calcein-positive cells (peak of excitation/emission 495/515) was performed under a fluorescence microscopy (Leica DMI3000, Nussloch, Germany). The quantification of cellular content in SF hydrogels was performed through a XTT cell proliferation assay based in colorimetry techniques (ATCC®; Cat#30-1011K). A total number of 1.5x10^4^ or 3x10^4^ mSCs were homogeneously resuspended in 50 µl of a previously sonicated silk fibroin solution and cultured on a 96-wells plate (BioLite, Thermo Scientific) until silk fibroin gelation was produced. Regular mSCs culture medium was added to each well to completely cover the hydrogel. At different time points after culture (12 hours, 72 hours, 1 week and 2 weeks) the culture medium was replaced with XTT and cells were incubated with this reagent for 4h at 37ºC. The measured absorbance at 450 nm, directly correlated to the number of viable cells, was determined on a microplate reader (BioTek ELX800).

*Tracking Studies*

Flow cytometry was used to quantify the EGFP fluorescence of mSCs derived from C57BL/6-Tg(CAG-EGFP) C14-Y01-FM131Osb mice. Flow cytometry acquisition was carried out in a FACSCalibur (Becton Dickinson; New Jersey, USA) on FL1 channel (excitation/emission at 488/530 nm). Gating of EGFP positive events and mean fluorescence intensity calculation were performed with FlowJo software package (FLOWJO, LLC, Ashland, OR). The magnitude of EGFP fluorescence was estimated at different passages after mSCs extraction and isolation from bone marrow. In vivo survival and retention of EGFP-expressing mSCs was examined by fluorescence microscopy and stereological analysis. At different time points (72 hours, two and four weeks) after striatal injection of 1x10^5^ EGFP-mSCs alone or enclosed into silk fibroin hydrogels the implanted animals were perfused transcardially with cold PBS followed by 4% paraformaldehyde under terminal anesthesia with chloral hydrate. The brains were removed, post-fixed for 48 h, cryoprotected in 30% sucrose and serially cut into 30 µm free-floating sections on a freezing microtome (Leitz Wetzlar). Coronal sections were mounted every 300 µm on poly-L-lysine-coated glass slides and covered with fluorescence mounting medium. The different images were captured with a fluorescence microscope (Leica DMI3000, Nussloch, Germany). The mSCS-EGFP content per section was calculated dividing the EGFP fluorescence area by the estimated area occupied by a single EGFP-cell, which in average was approximately 50-80 µm^2^. EGFP fluorescence areas were determined with imageJ software (NIH).

*Distal Middle Cerebral Artery Occlusion*

Focal cerebral ischemia was induced by direct occlusion of the distal part of the right middle cerebral artery (MCA) as previously described . Briefly, mice were anesthetized with 2 % isoflurane in air and a skin incision was made between the eye and ear under a dissection microscope. After the temporal muscle was separated, the MCA was identified through the semi-translucent skull and a burr hole (Ø 0.8mm) was made using a microdrill. After identification, the artery was permanently ligated using a nylon ophthalmic suture (9-0).

*Magnetic Resonance Imaging (MRI)*

This procedure was performed in mice as previously described with minor modifications . Briefly, 48 hours after focal ischemia MRI was performed in the C.A.I. Nuclear Magnetic Resonance and Electron Spin Center at Complutense University of Madrid using a BIOSPEC BMT 47/40 (Bruker, Ettlingen, Germany), which operates at 4.7 Teslas and is equipped with a 12 cm actively shielded gradient system. Under anesthesia with a mixture of oxygen and isoflurane the mice were injected intraperitoneally with 0.4 mmol/kg Gadopentetate dimeglumine (Gadolinium, Magnevist, Schering, Germany). Mice were placed in prone position on a plate, head immobilized and connected to a radiofrequency probe to monitor cardiac and respiratory vital signs. First optimal images in T2 were acquired using a fast spin echo sequence (acquisition parameters TR/TE=4000/60, FOV = 3 cm, slice thickness = 1 mm, matrix = 256 x 192). Then, T1 weighted spin echo images were acquired T1 (TR/TE=700/15 ms) using the same geometrical parameters as above.

*Infarct Volume Determination*

The infarcted area was determined as previously described . Brieﬂy, the mice were sacrificed by decapitation 72h after MCA ligation. The brains were removed and sliced in 1 mm thick coronal sections using a brain matrix (WPI, Florida, USA). The brain slices were immersed in a solution containing 1% of 2,3,5-triphenyltetrazolium chloride (TTC) in PBS. Coronal sections across the rostrocaudal axis were digitalized and the area of tissue damage delimited using the polygon selection tool of ImageJ (NIH). The volume of damaged tissue after infarction was calculated in relation to the contralateral (non-intact) hemisphere to correct for edema.

*Cortical Tissue Loss and Subcortical Expansion Measurement*

As a measure of brain necrosis, cortical tissue loss was determined at one and ten weeks after treatment. Under terminal anesthesia with chloral hydrate the animals were perfused transcardially with cold PBS followed by 4% paraformaldehyde. Brains were removed, post-fixed for 48 h and cryoprotected in 30% sucrose. The brains were serially cut into 30-μm free-floating sections on a freezing microtome. Coronal sections were mounted every 300-μm on poly-L-lysine-coated glass slides and stained with toluidine blue according to standard procedures. The images were captured with an Olympus BX51 microscope and Olympus DP70 camera. In both hemispheres, the area of interest was traced and measured with open source imageJ software (NIH). The percentage of cortical tissue loss in each section was calculated by: 100 - (remaining viable cortical area of infarcted hemisphere / cortical area of non-infarcted hemisphere x 100). The percentage of subcortical expansion in each section was estimated by: (subcortical area of infarcted hemisphere / subcortical area of non-infarted hemisphere x 100) -100.

*Behavioral Assessment*

Sensorimotor coordination was examined by the grid walking test . We analyzed the frequencies of slips with both forepaws during regular locomotion over a 13-mm square wire mesh. The animal behavior was videotaped and the total number of foot faults for each forelimb, along with the total number of non-foot-fault-steps was scored. A foot fault was considered positive when the respective forepaw slipped between the rungs or went through the grid hole causing the animal to lose balance. Blinded evaluation was examined on video recordings by L.F-G.

**2. Supplementary Figures**

**
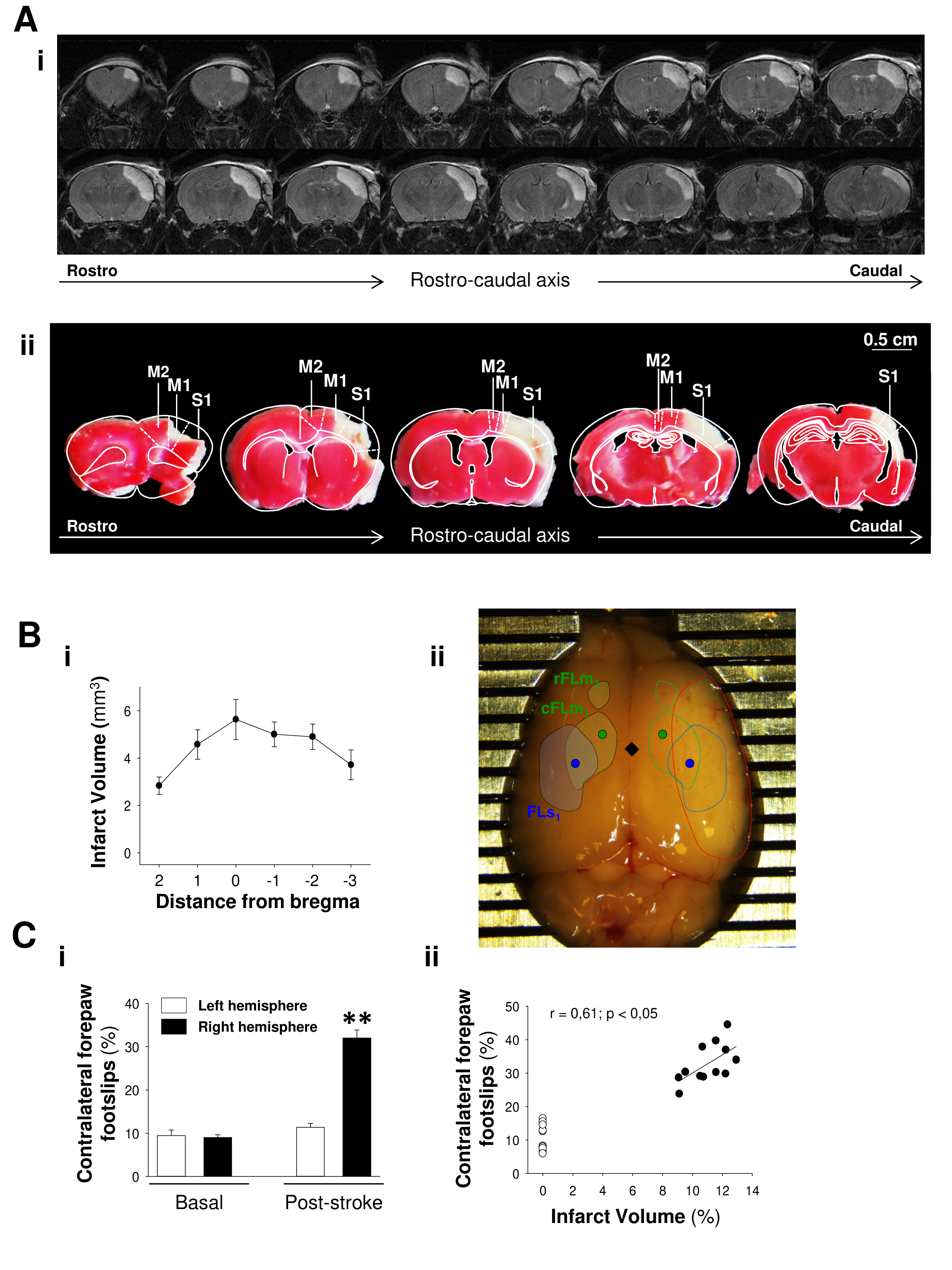
**

**Fig. S1. Brain damage and sensorimotor evaluation after middle cerebral artery occlusion.** (A) The top panel (i) shows representative diffusion-weighted MRI images of coronal sections illustrating the brain damage caused in the right hemisphere as a distinct hyperintense (white) area 48 hours after MCA occlusion surgery. The lower panel (ii) shows representative images of TTC-stained coronal sections 24 hours after MCA occlusion. The infarct area (in white) is mainly localized to cortical regions in areas affecting the somatosensory territory and, to a lesser extend, the motor cortex. (B) Panel i shows the regional distribution of the infarcted volume along the rostrocaudal axis as measured from bregma. Panel ii depicts a dorsal view of a mouse brain showing the affected territory in the right hemisphere (delimited with a dashed red line). The forelimb somatosensory and motor maps, as well as the position of electrodes for the recordings of evoked activity in FLs_1_ (damaged tissue) and cFLm_1_ (peri-lesional tissue) in infarcted and equivalent positions of the non-infarcted hemisphere are shown. (C) Panel i shows the left and right forepaw footslips examined with the grid walking test before the dMCAO surgery and one week after stroke. In this case, the left forepaw was contralateral to the right infarcted hemisphere. In the right panel (ii), Pearson’s correlation between left forepaw footslips and the volume of infarction (black symbols). The white symbols represent the left forepaw footslips before stroke. Data are presented as the means ± SEM from 12 mice. Black asterisks denote significant differences between footslips for the same forepaw before and after stroke (Student’s t-test; **p <0.01).

**
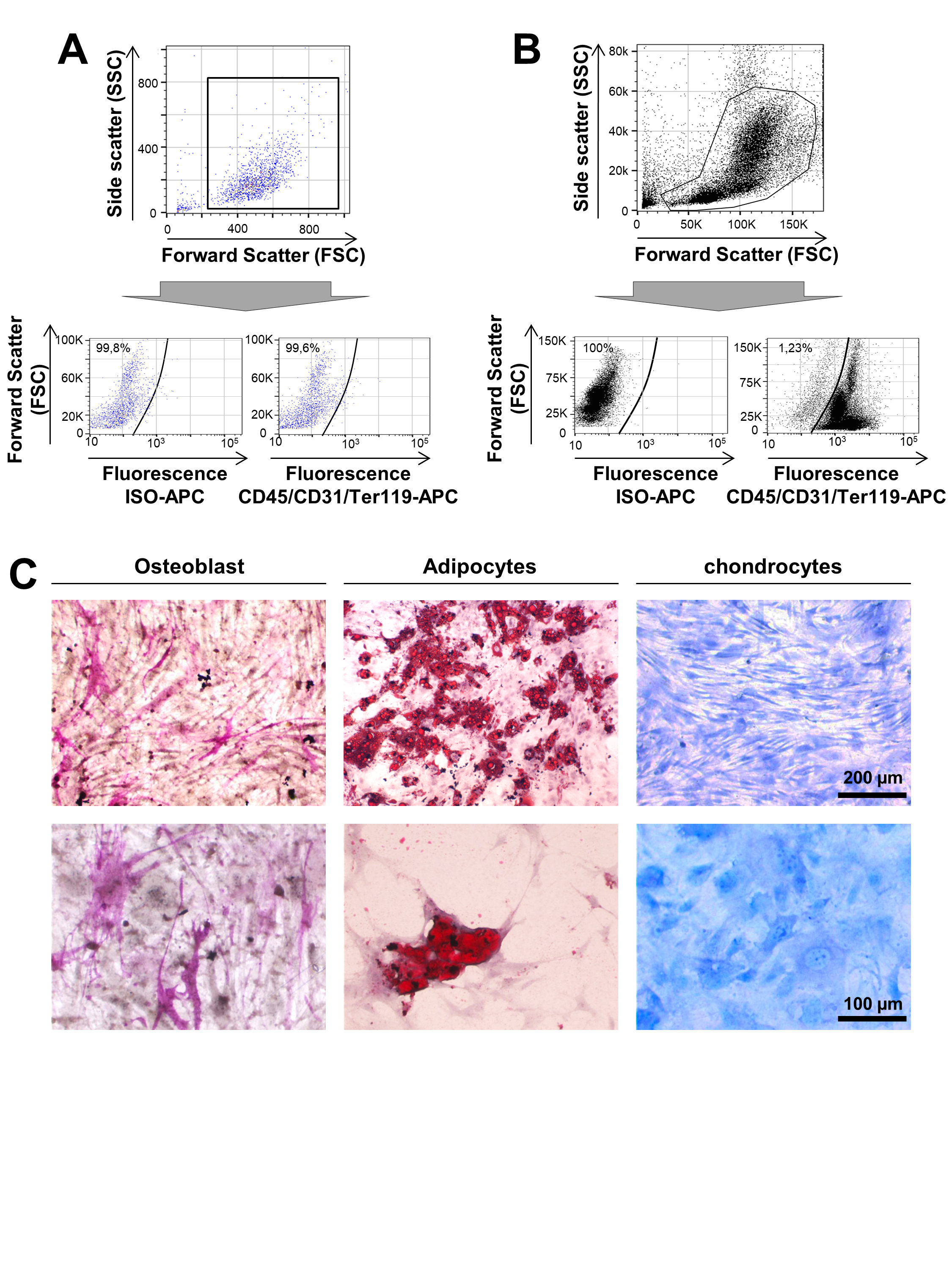
**

**Fig. S2. Phenotypic and functional characterization of mSCs mesenchymal stem/multipotent stromal cells.** (A) Top panel, forward scatter (cell size) versus side scatter (cell complexity) dot plot generated by flow cytometry from mSCs cultures expanded *ex vivo*. Bottom panel, this heterogeneous cell population was negative for cell surface markers present in hematopoietic (CD45), erythroid (Ter119) and endothelial cells (CD31). (B) Forward scatter vs side scatter dot plot from fresh bone marrow (BM), a tissue highly enriched in mSCs. The mSCs population represents a minor fraction (~1-1.5 %) of the total BM. (C) In vitro multipotent differentiation of mSCs into osteoblasts, adipocytes and chondrocytes, which constitute the “gold standard” assays for characterization of multipotent activity of mSCs.

**
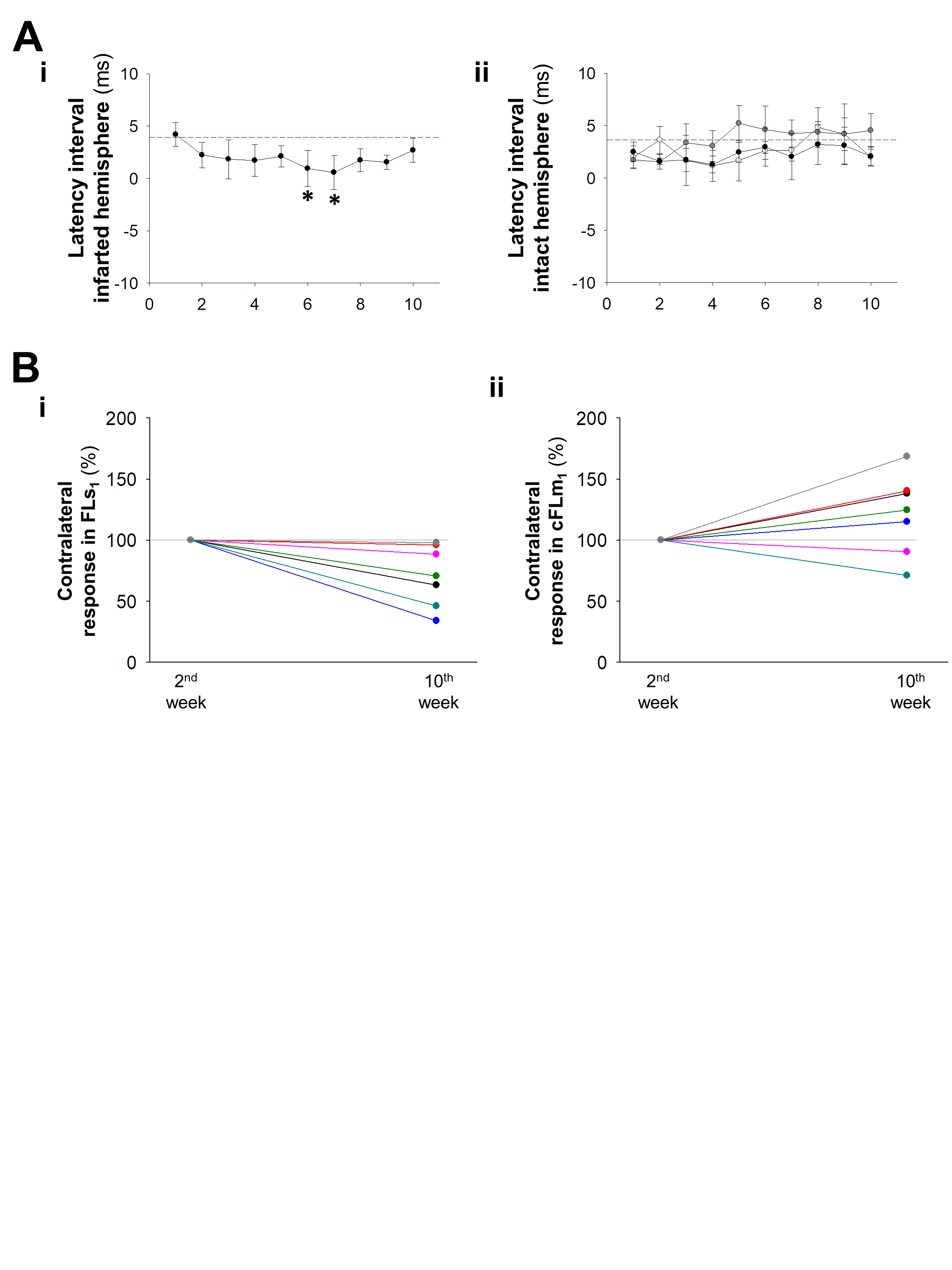
**

**Fig. S3. Latency interval between the maximal responses determined in FLs_1_ and cFLm_1_. Temporal evolution of evoked activity in FLs_1_ and cFLm_1_ at different time points after treatment.** (A) Latency interval for the infarcted (panel i) and non-infarcted hemispheres (panel ii) in mice treated with PBS (white circles), mSCs (gray circles) and mSCs encapsulated in silk fibroin hydrogels (black circles). (B) Percentage of contralateral evoked response in FLs_1_ and cFLm_1_ at 2 and 10 weeks post-treatment in mice implanted with mSCs encapsulated in silk fibroin hydrogels.

**Fig. S4. Intra-cortical connectivity in infarcted and non-infarcted hemispheres.** Pearson’s correlation coefficients between the maximal responses in FLs_1_ and cFLm_1_ for the infarcted and non-infarcted hemispheres in mice treated with PBS, mSCs or mSCs encapsulated in silk fibroin hydrogels.

**Fig. S5. Post-stroke cortical tissue loss and subcortical expansion after treatment with mSCs delivered into silk fibroin gels.** (A) Total cortical tissue loss in stroke animals at 96 hours after implantation (7 days post-stroke) of silk fibroin, mSCs or mSCs encapsulated in silk fibroin. The asterisks show significant differences between groups (one-way ANOVA test followed by Tukey’s test; **p<0.01). (B) Subcortical expansion (SE) 10 weeks after injection of PBS, mSCs alone or mSCs encapsulated in silk fibroin hydrogels. Left panel, subcortical expansion in coronal sections along the rostrocaudal axis as measured from bregma. The blue asterisk shows significant differences between mSCs and mSCs-SF mice (two-way ANOVA test followed by Tukey’s test; *p<0.05). Right panel, total subcortical expansion measured in the infarcted hemisphere (one-way ANOVA test followed by Tukey’s test; *p<0.05).

**
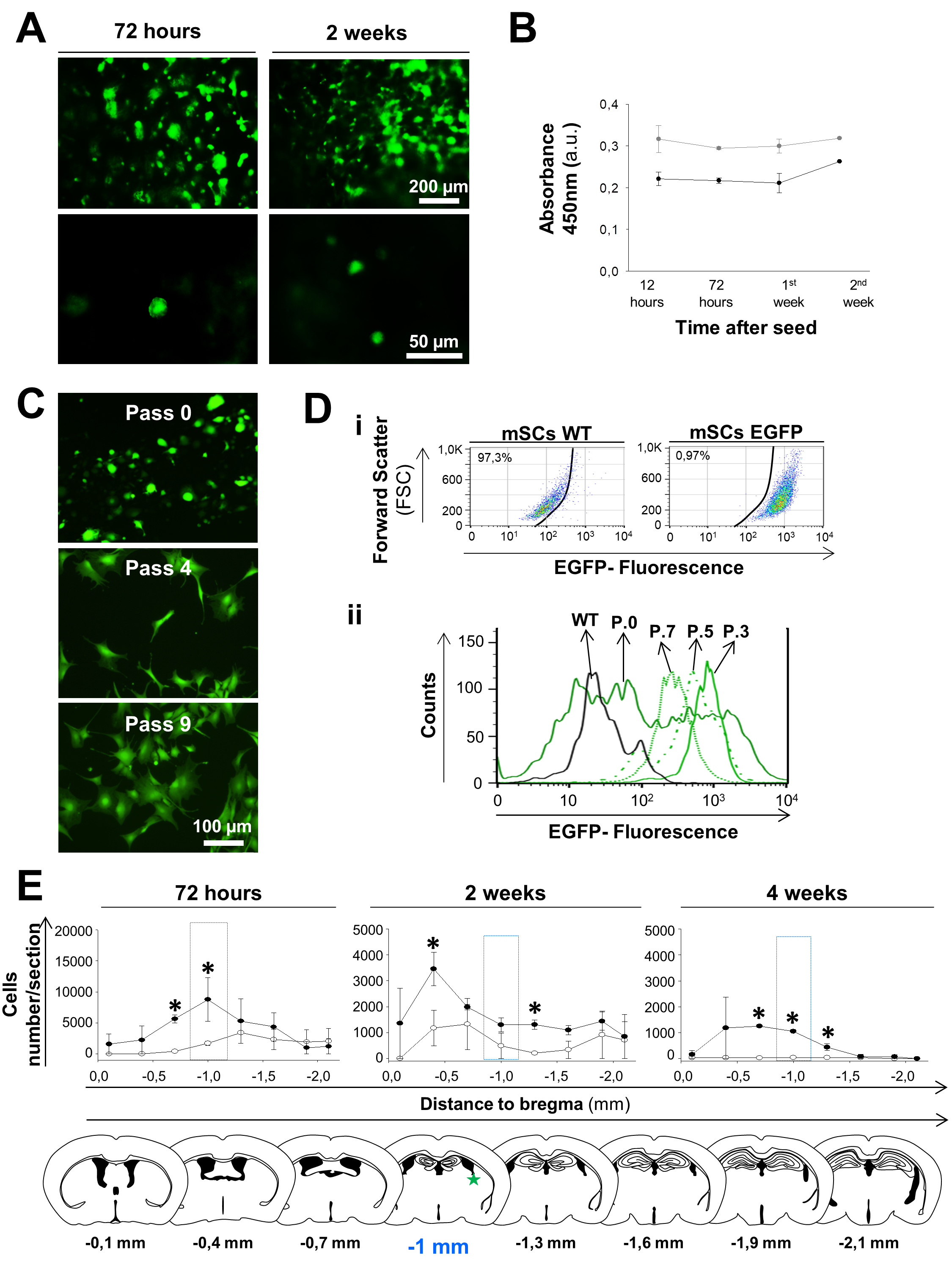
**

**Fig. S6. In vitro and in vivo survival of mesenchymal stem cells engrafted into silk fibroin hydrogels.** (A) Representative fluorescence microscopy images of viable calcein-positive mSCs integrated in silk fibroin hydrogels at 72 hours and two weeks of culture in vitro. (B) The XTT content measured at different time points after mSCs integration into the biomaterial indicates that the mSCs remain alive in the time period examined. In this study, two different cell densities were initially enclosed within the silk fibroin hydrogels (black circles: 15,000 cells; gray circles: 30,000). (C) Representative images of EGFP-expressing mSCs at different cell passages. (D) Panel i shows a representative dot plot of flow cytometry showing that the majority of mSCs were EGFP positive. Panel ii shows the mean fluorescence intensity of mSCs at different cell passages after culture. With the exception of mSCs derived from non-EGFP mice, the mSCs derived from EGFP-positive mice at passages P0, P3, P5, P7 show significant and relatively stable EGFP fluorescence. Note that P5-P7 was the range of passages used for the in vivo tracking experiments. (E) Comparative study of the survival and brain distribution of EGFP-expressing mSCs implanted alone (white circles) or encapsulated in silk fibroin hydrogels (black circles) *in vivo*. The number of cells per coronal section along the rostrocaudal axis as measured from bregma is shown at 72 hours and two and four weeks after implantation. The blue dashed rectangle indicates the site of injection in relation to the other coronal sections. Bottom panel, schematic showing examples of the coronal brain sections analyzed based on Paxinos & Franklin Atlas and the site of mSCs implantation (green star). Data are shown as the means ± the SEM from a minimum of 6-8 mice per group (mSCs or mSCs-silk fibroin) at each temporal point. In each coronal section analyzed the asterisks show significant differences between groups (Two-way ANOVA test followed by Tukey’s test; *p<0.05).

**REFERENCES**

Barios, J. A., Pisarchyk, L., Fernandez-Garcia, L., Barrio, L. C., Ramos, M., Martinez-Murillo, R., et al., (2016). Long-term dynamics of somatosensory activity in a stroke model of distal middle cerebral artery oclussion. J Cereb Blood Flow Metab. 36**,** 606-620. 10.1177/0271678X15606139

Gonzalez-Nieto, D., Li, L., Kohler, A., Ghiaur, G., Ishikawa, E., Sengupta, A., et al., (2012). Connexin-43 in the osteogenic BM niche regulates its cellular composition and the bidirectional traffic of hematopoietic stem cells and progenitors. Blood. 119**,** 5144-5154. 10.1182/blood-2011-07-368506

Martinez-Murillo, R., Martinez, A., (2007). Standardization of an orthotopic mouse brain tumor model following transplantation of CT-2A astrocytoma cells. Histol Histopathol. 22**,** 1309-1326. 10.14670/HH-22.1309
